# Parallel analysis of *Arabidopsis* circadian clock mutants reveals different scales of transcriptome and proteome regulation

**DOI:** 10.1098/rsob.160333

**Published:** 2017-03-01

**Authors:** Alexander Graf, Diana Coman, R. Glen Uhrig, Sean Walsh, Anna Flis, Mark Stitt, Wilhelm Gruissem

**Affiliations:** 1 Department of Biology, ETH Zurich, 8092 Zurich, Switzerland; 2 Max Planck Institute of Molecular Plant Physiology, 14476 Postdam-Golm, Germany

**Keywords:** circadian clock, photoperiod, transcriptomics, proteomics, *Arabidopsis thaliana*

## Abstract

The circadian clock regulates physiological processes central to growth and survival. To date, most plant circadian clock studies have relied on diurnal transcriptome changes to elucidate molecular connections between the circadian clock and observable phenotypes in wild-type plants. Here, we have integrated RNA-sequencing and protein mass spectrometry data to comparatively analyse the *lhycca1*, *prr7prr9*, *gi* and *toc1* circadian clock mutant rosette at the end of day and end of night. Each mutant affects specific sets of genes and proteins, suggesting that the circadian clock regulation is modular. Furthermore, each circadian clock mutant maintains its own dynamically fluctuating transcriptome and proteome profile specific to subcellular compartments. Most of the measured protein levels do not correlate with changes in their corresponding transcripts. Transcripts and proteins that have coordinated changes in abundance are enriched for carbohydrate- and cold-responsive genes. Transcriptome changes in all four circadian clock mutants also affect genes encoding starch degradation enzymes, transcription factors and protein kinases. The comprehensive transcriptome and proteome datasets demonstrate that future system-driven research of the circadian clock requires multi-level experimental approaches. Our work also shows that further work is needed to elucidate the roles of post-translational modifications and protein degradation in the regulation of clock-related processes.

## 1. Introduction

The presence of a circadian clock in organisms from across the domains of life reflects its fundamental importance for organismal growth and survival [1,2]. The circadian clock in plants coordinates and fine-tunes various cellular pathways to optimize growth in relation to internal (e.g. sugar) and external (e.g. light) cues [3]. At the molecular level, the circadian clock establishes a state of cellular preparedness through the precise regulation of gene expression in anticipation for the transitions during the light–dark cycle [2,4,5].

The plant circadian clock comprises genes encoding proteins that establish a series of interlocked negative feedback loops, which form a regulatory oscillator [6,7]. In *Arabidopsis thaliana* (*Arabidopsis*), the clock proteins forming the core of these negative feedback loops include transcriptional regulators acting as repressors or activators that organize into the ‘morning’ and ‘evening’ loops [6,7]. The ‘morning loop’ comprises the genes encoding CIRCADIAN CLOCK ASSOCIATED 1 (CCA1) and LATE ELONGATED HYPOCOTYL (LHY), which increase their expression prior to dawn [8,9], while the ‘morning loop’ genes encoding PSEUDO-RESPONSE REGULATOR (PRR) 9, 7 and 5 sequentially increase their expression after dawn [10,11]. The ‘evening loop’ comprises genes encoding GIGANTEA (GI) and TIME OF CAB EXPRESSION 1 (TOC1) [12–14] as well as the evening complex genes encoding EARLY FLOWERING (ELF) 3, 4 and LUX ARRHYTHMO (LUX) [15–17], which increase their expression prior to, and after dusk. The ‘morning’ and ‘evening’ loop proteins regulate each other through a series of promoter *cis*-elements [18–20] and protein–protein interactions [2,17,21,22], creating a robust oscillator that regulates gene expression in a coordinated 24 h rhythm. However, our knowledge of the extent of circadian clock control over downstream components remains incomplete. To address this at the systems level requires parallel analysis of transcriptome and/or proteome changes in multiple clock mutants.

The circadian clock regulates a number of key physiological and developmental processes in plants including metabolism [23–25], leaf movement [26], flowering [8,22], growth [17], hormone levels [27,28] and stress responses [27], among others [2,5,29]. Many of these roles were identified and characterized in clock mutant plants, which continue to be central to understanding the clock and the processes it controls [2]. The plant clock can be entrained by a number of interrelated external and internal cues, such as light [30] and temperature [31,32], as well as photosynthesis and metabolism [33], which together function to ‘set the timer’ and to synchronize the internal clock with the external photoperiod. Although the mechanisms communicating these cues to the circadian clock are not fully understood, they result in tissue-dependent [34] and/or cell-type-dependent [35] response-adjusted expression of output genes through the transcription factors (TFs) and repressors of its ‘morning’ and ‘evening’ loops [6,19,20]. Clock-controlled gene expression is further regulated through the modification of chromatin by ubiquitination [36,37], acetylation [38,39] and methylation [40–42]. Context-dependent stability (e.g. light stability and dark instability) of core clock components [43–45] and output proteins also plays a key role in clock function and signalling [46,47]. Furthermore, the core circadian clock proteins LHY and CCA1 [48–50] as well as TOC1, PRR3 and PRR5 [51] are regulated by reversible phosphorylation, as are proteins involved in clock-regulated input/output processes, such as starch metabolism [52] and isoprenoid biosynthesis [53]. Despite known roles for PTMs in fine-tuning the clock in organisms such as *Drosophila* [1,54], our understanding of how these modifications impact the regulation of clock components and its input/output processes is still limited in plants [2,55,56].

The phenotype of mutations in the genes encoding the core clock components *LHY/CCA1*, *PRR7/PRR9*, *GI* and *TOC1* have been reported. In *Arabidopsis*, loss of LHY/CCA1 function results in a short-photoperiod phenotype with a forward shift in peak gene expression and rapid loss of rhythmicity [57]. PRR7/PRR9 loss of function mutants have a long photoperiod phenotype with delayed peak gene expression and rapid loss of rhythmicity [10]. Depending on the allele, *Arabidopsis* GI mutants have multiple phenotypes, including short- or long-photoperiod phenotypes with perturbed gene expression amplitude [58]. Similarly, loss of TOC1 function results in a short-period phenotype together with a perturbed gene expression amplitude [59].

Considering that a large proportion of the *Arabidopsis* genome is under transcriptional control by the clock [19,60], the majority of reports investigating biological processes associated with the clock in plants have focused on changes in the transcriptome [2,5,20]. However, recent studies have demonstrated a disconnect between fluctuations in global gene transcription and the corresponding proteome [61–65]. To further understand the function of the circadian clock and processes regulated by its core proteins we used high-throughput RNA sequencing (RNA-seq) and quantitative protein mass spectrometry to analyse global transcript and protein level changes in *Arabidopsis* rosettes from circadian clock mutants harvested at end of day (ED) and end of night (EN). Loss of circadian clock proteins indicates a modular regulation where each clock loop predominantly regulates a different set of genes at the transcript and protein level involving different subcellular compartments. Our results also show that integrating proteome analysis in clock studies significantly advances our understanding of the control that the circadian clock exerts on input/output processes.

## 2. Results and discussion

**Figure 1.**
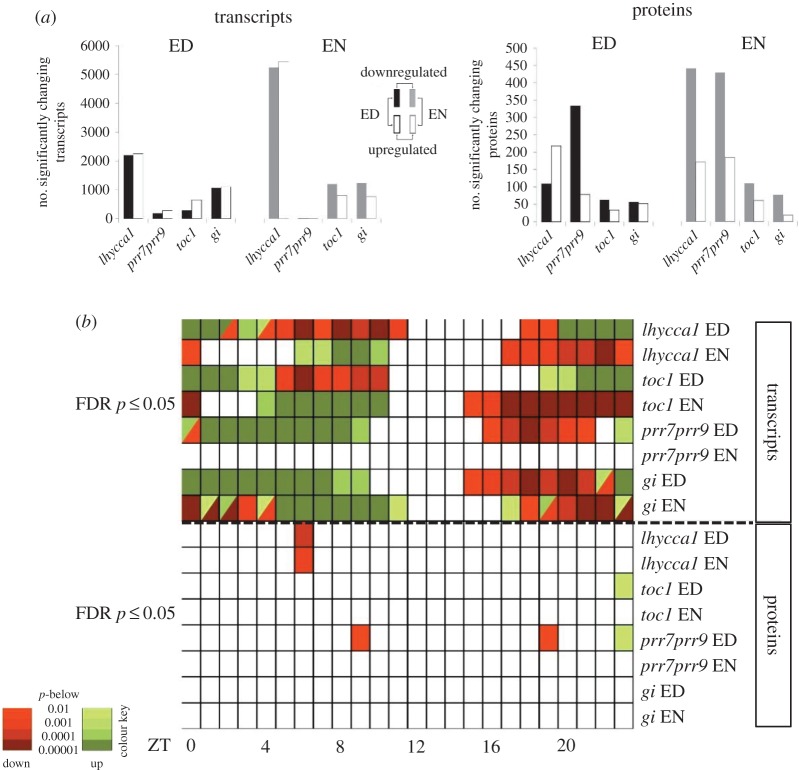
Loss of core circadian clock genes impacts the transcriptome and proteome of *Arabidopsis thaliana*. (*a*) Number of significantly changing transcripts and proteins (FDR-adjusted *p* ≤ 0.05). (*b*) Over-representation of ZT peak expression of changing transcripts and proteins (relative to peak diurnal transcript expression) identified in each clock mutant at ED and EN (fisher's exact test: FDR *p* ≤ 0.05). Colours represent over-representation of ZTs in upregulated (green) or downregulated (red) transcripts (upper panel) or proteins (lower panel). Darker colours indicate lower *p*-values. Diurnal transcript expression data were obtained from Diurnal DB.

Using whole rosettes from *Arabidopsis* circadian clock mutants *lhycca1*, *prr7prr9*, *gi* and *toc1*, we analysed, in parallel, mutant-induced changes in transcript and protein abundance before day–night transitions: 1 h (h) before ED and EN. The four mutants have well-defined impacts on the circadian clock. Therefore, by examining growing rosette leaves harvested ED and EN we aimed to capture time points important for both clock regulation before the light/dark transition and plant metabolism before the transition from the autotrophic to the heterotrophic phase. Collectively, among the 17 934 transcripts and 3169 proteins consistently identified and quantified between each mutant and photoperiod time point, our data reveal extensive changes in both transcript and protein abundance (FDR-adjusted *p* ≤ 0.05; figure 1*a*; electronic supplementary material, tables S1–S2). The transcript and protein changes in the clock mutants were analysed considering also differences in the transcriptomes and proteomes of the Col-0 and Ws-2 ecotypes (electronic supplementary material, figure S1, tables S3–S5).

### 2.1. Loss of core circadian clock components affects both the transcriptome and proteome of *Arabidopsis*

The majority of differentially expressed genes (DEGs) were observed in *lhycca1*, where 4448 genes ED and 10 478 genes EN showed either significant increase or decrease in transcript abundance (figure 1*a*; electronic supplementary material, figure S2 and table S1). Mutants *prr7prr9*, *gi* and *toc1*, however, had only 460, 2166 and 943 significant DEGs at ED, and 16, 1995 and 1995 at EN, respectively. Analysis of each mutant at ED and EN revealed significant over-representation of both circadian clock-regulated and diurnally regulated genes (Diurnal DB, http://diurnal.mocklerlab.org; electronic supplementary material, table S6). Changes in mutant proteomes were less pronounced, however. The majority of the changes in protein abundance were observed in *lhycca1* and *prr7prr9* ED (328, 413) and EN (633, 614) (figure 1*a*; electronic supplementary material, table S2). A significant over-representation of proteins encoded by either clock regulated or diurnally-regulated genes was observed for all mutant lines (electronic supplementary material, table S6). Hence, the loss of core circadian clock components causes significant changes in both transcriptomes and proteomes that are highly enriched for circadian and diurnally regulated genes.

We further analysed the DEGs and protein level changes in relation to known Zeitgeber (ZT) peak transcript expression to determine if changes in the expression of clock-regulated genes were over-represented for specific ZTs in the diurnal cycle [66] (Diurnal DB). In the *lhycca1* mutant, upregulated DEGs with peak expression between ZT20 and ZT4 were over-represented at ED while for downregulated DEGs peak expression between ZT4 and ZT10 as well as ZT18 and ZT19 was over-represented (figure 1*b*). At the EN time point, the observed over-representation of peak expression ZTs in *lhycca1* was inverted compared with ED. These results indicate that genes with specific peak expression phases are preferentially affected in this mutant. This could be attributed to the ‘short-period’ phenotype of the *lhycca1* mutant that leads to a shift in expression phase of clock-regulated genes. At ED (ZT12), morning-phased genes are already induced and consequently identified as upregulated while genes with a peak expression during the afternoon or early night already passed their expression peak and are identified as downregulated.

Consistent with this finding, *toc1,* the second ‘short-period’ mutant we analysed, showed a similar pattern of over-represented peak expression ZTs as *lhycca1* [57,67]. This pattern in *prr7prr9* and *gi* mutants was comparable at ED. While *prr7prr9* has a long-period phenotype [10,68], the *gi* mutant we used showed no difference in clock period compared with WT except that the amplitude of expression of core clock genes and clock-regulated genes is dampened in the *gi* mutant [58]. However, the analysis of peak expression ZTs alone cannot answer whether the observed over-representation patterns are due to a shift in phase or changes in the amplitude of clock-regulated transcripts. Unlike the transcriptome, proteome changes had minimal significant over-representation of ZT peak expression, indicating that a shift in the phase or reduction in amplitude of circadian controlled transcripts has no strong effect on the measured proteome (figure 1*b*; electronic supplementary material, table S6).

**Figure 2.**
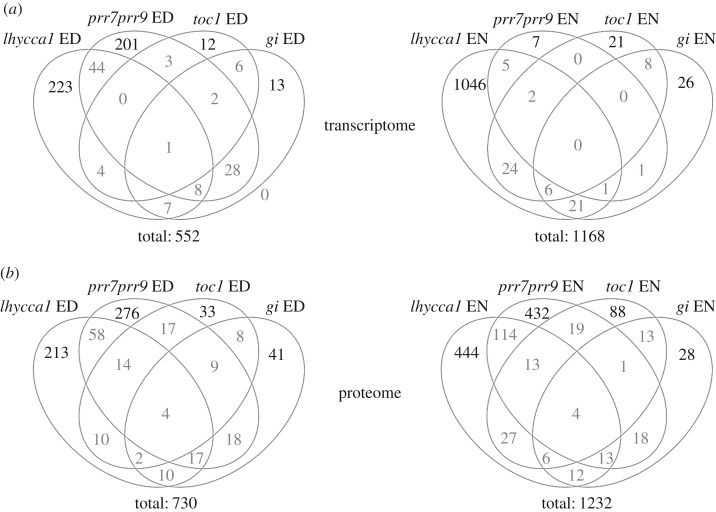
Loss of circadian clock genes differentially impacts the transcriptome and proteome. (*a*) Venn diagrams depict the overlap of genes showing a significant transcriptional change in abundance greater than 1.5-fold and FDR *p* ≤ 0.05 relative to their corresponding wild-type background. (*b*) Venn diagrams showing the overlap of genes encoding proteins whose levels are significantly different in each mutant compared with their respective wild-type background (FDR *p* ≤ 0.05). Specific deregulation (upregulation or downregulation) at the ED and EN time points are shown.

**Figure 3.**
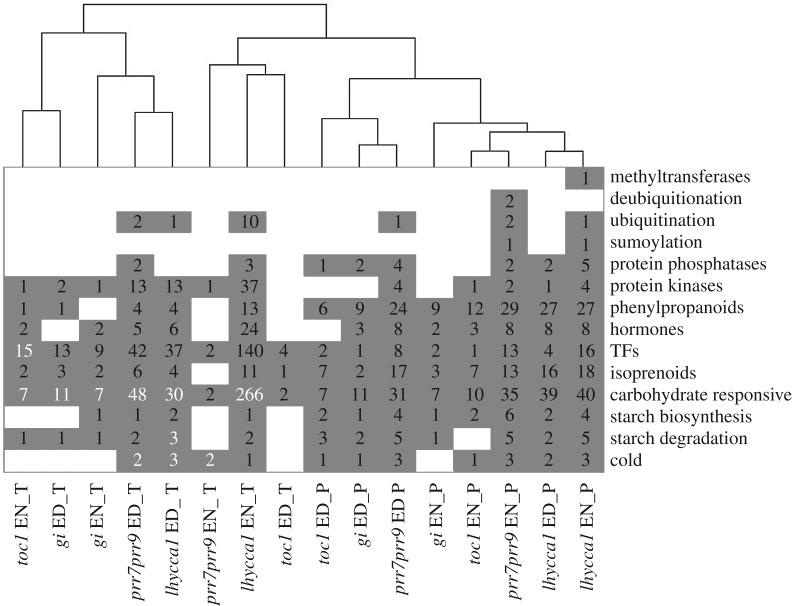
Over-representation of genes exhibiting a statistically significant change in transcript and/or protein abundance. Depicted are the number of significantly changing transcripts (FDR *p* ≤ 0.05 and FC > 1.5) and proteins (FDR *p* ≤ 0.05) within each category. Values in each box denote the number of significantly changing genes, while those values highlighted in white are significantly over-represented (Fisher's test; *p* ≤ 0.05). Hierarchal clustering was performed using Heatmap.2 with a Euclidean distance measure and the ‘complete’ hierarchical clustering agglomeration method.

Using FDR-adjusted *p* ≤ 0.05 and FC ≥ 1.5 for significantly changing transcripts and FDR *p* ≤ 0.05 for proteins we calculated the overlap between mutants with respect to the changes in transcript and protein levels. We found that changes of both transcript and protein levels were largely specific for each mutant (figure 2). Furthermore, we analysed metabolic pathways and cellular processes that are affected by mutations of core clock genes. We compiled categories representing metabolic pathways and functional processes based on the top 25 enriched GOs across the clock mutants (see Material and methods). Next, we analysed the distribution of significantly changing transcripts and proteins within these categories and identified specific over-representations (electronic supplementary material, table S7). The highest number of DEGs was observed in the categories ‘carbohydrate responsive’, ‘TFs’ and ‘protein kinase’ across all mutants (figure 3; electronic supplementary material, table S7). In all mutants, DEGs were statistically over-represented in the ‘carbohydrate responsive’ category (figure 3; electronic supplementary material, table S7)*.* At the protein level, the highest number of changes in abundance was observed in the categories ‘carbohydrate responsive’, ‘TFs’, ‘isoprenoids’ and ‘phenylpropanoids’. However, no statistically significant over-representation of any category was observed at the protein level.

### 2.2. Genes involved in carbohydrate metabolism are affected in all clock mutants

The ‘carbohydrate responsive’ category is of interest because genes belonging to this category respond specifically to the availability of carbohydrates [69], which is a key consideration for ED versus EN. For *lhycca1* mutants it was expected that many changes of transcripts and proteins fall into this category. This double mutant has a shortened clock period leading to the anticipation of dawn 3–4 h before the actual EN [18]. This in turn leads to accelerated starch degradation during the night and carbon limitation before dawn [23,25,70]. Unexpectedly, our data indicate that carbohydrate responses are not restricted to *lhycca1* but are found in all mutants and at all time points (figure 3). To analyse these responses in more detail, we queried our data to further separate the ‘carbohydrate responsive’ category into carbohydrate-induced and carbohydrate-repressed genes [69].

**Figure 4.**
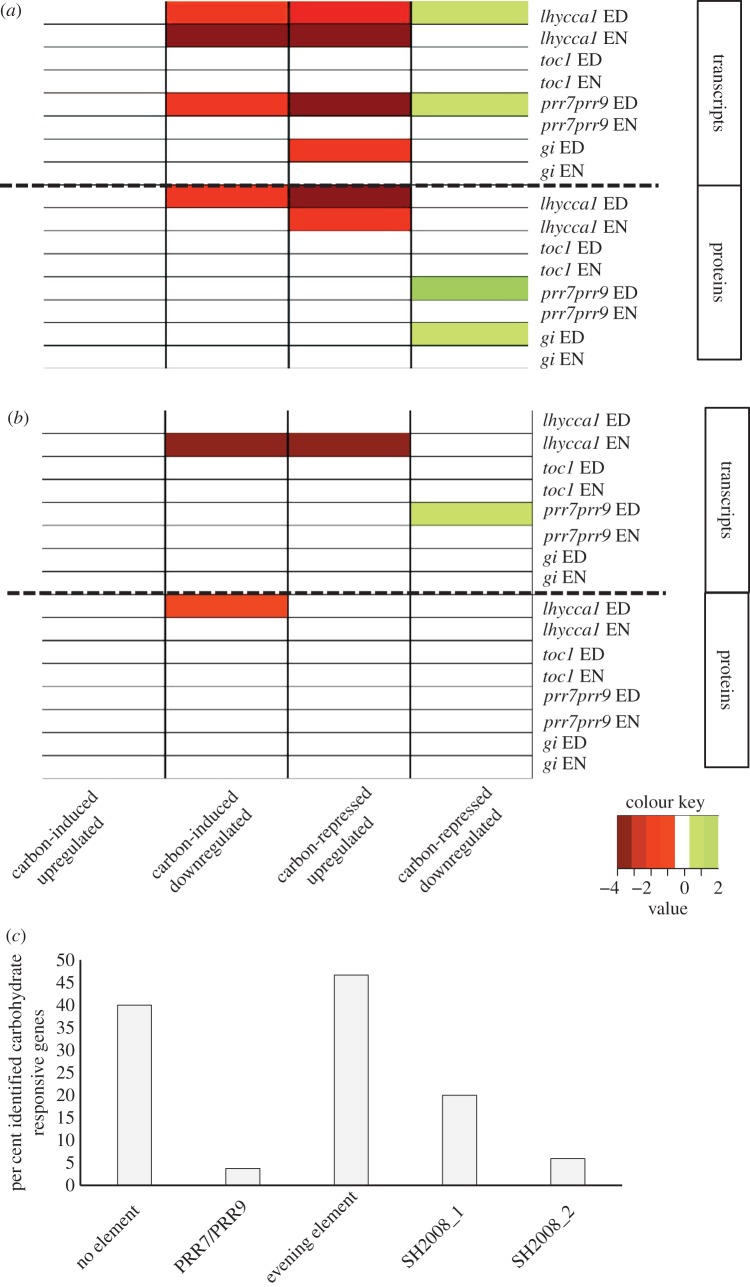
Over-representation of carbohydrate-induced and repressed genes showing significant change in clock mutants at the transcript and protein level. (*a*) Analysis of carbohydrate-induced and repressed genes as defined by Usadel *et al*. [69]. (*b*) Analysis using a refined, clock-independent set of carbohydrate responsive genes. Significant enrichment was determined using a Fisher's exact test (*p* ≤ 0.05). (*c*) Known circadian clock *cis*-regulatory elements encoded by carbohydrate-induced and repressed genes as defined by Usadel *et al*. [19,68,69,71]. Colours represent responses indicative of lower (red) and higher carbohydrate level (green) relative to each respective wild-type. Darker colours represent lower *p*-values.

Carbohydrate responsive genes were over-represented in *lhycca1*, *prr7prr9* and *gi* at both the transcript and protein level (figure 4*a*,*b*). This was most striking in *lhycca1* and *prr7prr9* at ED. At this time point, DEGs and changes in protein abundance in both mutants showed enrichment in carbohydrate responsive genes indicative of carbon starvation and carbon excess (figure 4*a*, red and green colour, respectively). To explain this result, we analysed the genes categorized as carbohydrate responsive by Usadel *at al*. [69] and found a strong enrichment of clock-regulated genes. While 18% of all transcripts quantified in this study are clock-regulated, 23.7% of all carbohydrate-induced genes (Fisher's exact test *p* *=* 2.0 × 10^−3^) and 54% of the carbohydrate-repressed genes (Fisher's exact test *p* *=* 1.0 × 10^−51^) also fall into this category. This large percentage indicates extensive transcriptional regulation of carbohydrate responses by the circadian clock. Promoter analysis of these carbohydrate responsive genes changing at the transcript level revealed that 60.3% had at least one known circadian clock *cis*-regulatory element in their promoters (figure 4*c*; electronic supplementary material, table S8) [19,68,71]. To further refine the analysis of the carbohydrate-related responses in our data, we assembled a set of clock-independent carbohydrate responsive genes by removing clock-regulated genes from the gene set published by Usadel *et al*. [69]. Using the resulting subset, most of the previously observed enrichments of carbohydrate-related responses were not recovered, indicating that they were indeed clock-regulated genes in our original list of carbohydrate responsive genes (figure 4*a*,*b*). A strong enrichment of carbohydrate-related responses indicative of carbon starvation was only observed in the DEGs of *lhycca1* at EN (figure 4*b*), which is consistent with previous reports on the phases of *lhycca1* carbon starvation during the night [23,70].

### 2.3. Changes in the expression of genes for protein phosphorylation and ubiquitination enzymes suggest a role for post-translational protein regulation

Considering that post-translational protein modifications are central to cell function and differentially expressed protein kinase genes were over-represented in our data, we investigated genes encoding the subsets of enzymes responsible for catalysing post-translational modifications (PTMs) in the clock mutants. A total of 60 genes encoding protein kinases had significant transcript level changes in the clock mutants (electronic supplementary material, table S9). Nine genes encoding ubiquitin ligases also showed significant changes in either transcript or protein abundance (electronic supplementary material, table S9). No changes in genes encoding enzymes for protein acetylation or methylation were found. The numbers of protein kinases and ubiquitin ligases that had altered transcript and/or protein levels were higher in both *lhycca1* and *prr7prr9* than *toc1* (figure 3).

Eleven of the 60 protein kinase genes with transcriptional changes in the clock mutants encode either sucrose non-fermenting-related (SnRK) or calcineurin B-like (CBL) interacting kinases (CIPKs), while the others encode uncharacterized leucine-rich repeat (LRR) or LRR-like and cysteine-rich receptor-like (RLK) protein kinases (electronic supplementary material, table S9). Promoter analysis of these protein kinases changing at the transcript level showed that 58.3% had at least one known circadian clock *cis*-regulatory element in their promoters (electronic supplementary material, figure S3 and table S9) [19,68,71]. SnRK family kinases regulate cellular processes ranging from stress [72]. In particular, SnRK1-family kinase 1.1 (SnRK1.1) directly regulates isoprenoid pathway flux, which is central to hormone production (e.g. brassinosteroids and cytokinins) through HMG-CoA Reductase (HMGR) [53]. SnRK1-family kinases have also been proposed as mediators that connect starch breakdown at night to clock regulation of carbohydrate reserves [73], while SnRK2.3 maintains circadian regulated phosphorylation paralleling its circadian regulated transcript level [55]. CIPKs decode intracellular Ca^2+^ signals [74], including the circadian oscillation of Ca^2+^ concentrations [75,76]. The changes in transcript abundance of CIPKs suggest their involvement in clock-related processes, similar to mammals in which Ca^2+^-regulated calmodulin kinase II (CaMKII) functions in clock regulation [77,78].

E3 ubiquitin ligases involved in the proteasome degradation pathway had either transcript or protein level changes only in *lhycca1* and *prr7prr9* (figure 3). Promoter analysis of these protein ligases and associated proteins changing at the transcript level found that 83.3% maintained at least one known circadian clock cis-regulatory element in their promoters (electronic supplementary material, figure S3 and table S9) [19,68,71]. FLAVIN-KELCH-FBOX-1 (FKF1; At1g68050) transcript levels changed most dynamically with a 5.9-fold increase at EN and 1.6-fold decrease at ED in *lhycca1* (electronic supplementary material, table S1 and S9). FKF1 is related to the clock protein ZEITLUPE (ZTL) [79], and forms a blue light-induced complex with GI to coordinate plant flowering time [39,80]. Five RING-family E3 ubiquitin ligases (At1g49210, At4g03510, At4g11360, At4g28270 and At5g27420) also had significant transcript level changes (electronic supplementary material, table S1 and S9), and three of these (At4g03510, At4g11360, At4g28270) are likely to be involved in carbon starvation response regulation [69]. Transcripts of the E3 ligase CARBON/NITROGEN INSENSITIVE 1 (CNI1; At5g27420) were upregulated in *lhycca1* at EN and decreased at ED (electronic supplementary material, table S1 and S9), coincident with the enrichment of carbohydrate responsive genes responding to carbon starvation of *lhycca1* plants at EN. CNI1 is a plasma membrane bound protein [81] that integrates signals from carbon and nitrogen supply through members of the 14-3-3 protein gene family, and thus is a key protein connecting the clock to plant growth [82,83].

### 2.4. Circadian clock regulation of gene expression is modular

Analysis of transcriptome changes in each clock mutant suggests that clock regulation at the transcript level is modular, with each clock component controlling a specific gene set (figure 2*a*). Concurrent examination of *lhycca1*, *prr7prr9*, *toc1* and *gi* clock mutants revealed only a small overlap of similarly regulated genes. Specifically, there are three distinct groups of genes that were similarly changed between *lhycca1* and *prr7prr9* at ED (44 genes), *gi* and *prr7prr9* at ED (28 genes), and *toc1* and *lhycca1* at EN (24 genes) (figure 2*a*; electronic supplementary material, table S10). In these overlapping DEGs only few metabolic pathways or functional processes were over-represented, such as cold responsive genes in *lhycca1* and *prr7prr9* at ED or carbohydrate responsive genes in *toc1* and *lhycca1* at EN and *gi* and *prr7prr9* at ED (electronic supplementary material, table S10). This latter result can be attributed to the overlap with clock-regulated genes in the ‘carbohydrate responsive’ gene category as discussed above.

Genes and proteins involved in PTM also appear to be regulated in a modular fashion by the circadian clock. Among more than 50 DEGs encoding protein kinases and phosphatases changing across the clock mutants, only three genes encoding kinases (At5g45820: CIPK20, At3g45860: CRK4, At4g23200: CRK12) and one gene encoding a protein phosphatase (At4g27800) were similarly changed in an overlapping gene set (figure 2; electronic supplementary material, table S10–11). Thus, each circadian clock component regulates a distinct set of kinases and phosphatases at the transcriptional level, and therefore likely the phosphorylation of distinct groups of clock-regulated proteins. LHY and CCA1 [49,50,84] as well as TOC1, PRR7, PRR5 and PRR3 [85,86] are all phosphorylated by either casein kinase II (CKII) or yet unknown protein kinase(s) to regulate protein stability or modify protein–protein interactions [86]. Only the expression of *CIPK20* was upregulated in both *gi* and *prr7prr9*, while genes encoding the receptor-like kinases *CRK4* and *CRK12* changed in *lhycca1* and *prr7prr9* (−1.9-fold/1.7-fold, respectively) at ED*.* The functions of CIPK20, CRK4 and 12 are currently unknown. The only protein phosphatase for which we could measure changes in protein levels in both *lhycca1* and *prr7prr9* at ED is the thylakoid-associated protein phosphatase 38 (TAP38), a PP2C phosphatase. TAP38, which specifically dephosphorylates the light harvesting complex II, is central for light harvesting and signalling [87]. TAP38 transcript levels are both diurnal and circadian regulated (Mockler Lab DB), and the *Arabidopsis* *tap38* mutant maintains rapid growth under constant light [88]. Together, the gene expression changes we found for protein kinases between the circadian clock mutants, as well as multiple proteins that have circadian regulated protein phosphorylation changes [55], indicate that protein phosphorylation is central for regulating clock-related processes. By contrast, no changes were found for protein ubiquitination or de-ubiquitination enzymes among the overlapping gene sets.

### 2.5. Transcriptome and proteome responses to circadian clock perturbation affect cellular compartments differently

**Figure 5.**
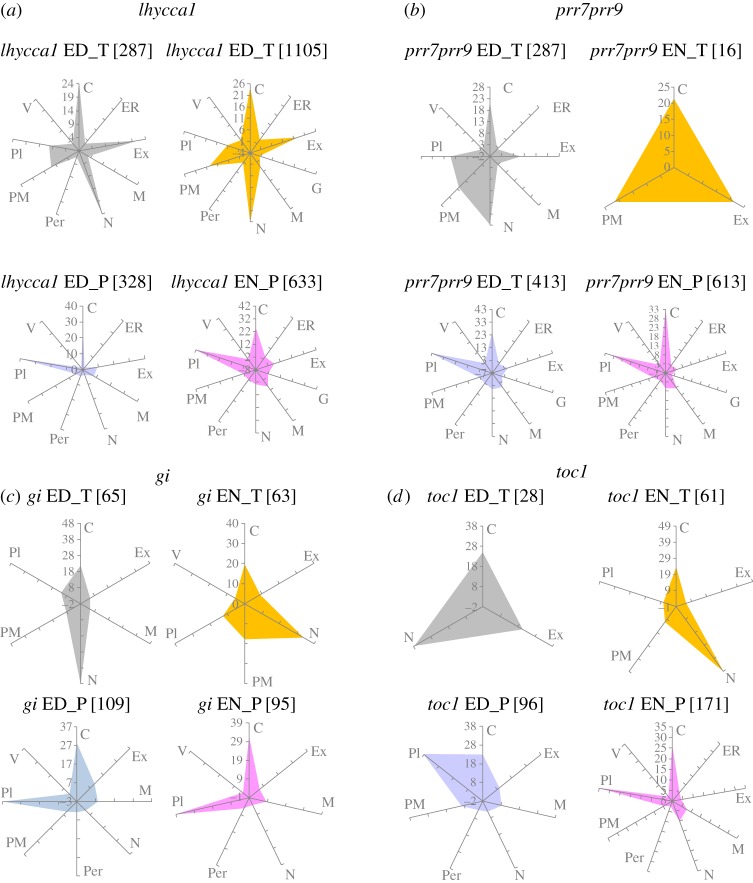
Subcellular localization of the changing transcriptome and proteome from circadian clock mutants ED and EN. (*a–d*) Radar plots illustrate the percentage of changing transcripts or proteins ED or EN occupying a specific subcellular localization for each clock mutant. Total number of changing genes included in each radar plot is shown in brackets. Grey, orange, blue and pink depict significantly changing transcripts ED, transcripts EN, protein ED and protein EN, respectively. C, cytosol; ER, endoplasmic reticulum; Ex, extracellular; G, Golgi; M, mitochondria; N, nucleus; Per, peroxisome; PM, plasma membrane; Pl, plastid; V, vacuole.

We used SUBAcon to predict the subcellular localization of proteins encoded by DEGs and proteins whose accumulation was changed in the clock mutants [89] (figure 5; electronic supplementary material, table S12–13). All organelles and compartments were affected, although the extent to which changes in the specific mutant transcriptomes or proteomes are related to any given cell compartment varies among the clock mutants. In particular, transcript changes in *prr7prr9* could affect proteins of all subcellular localizations at ED but only the endoplasmatic reticulum (ER), extracellular and plasma membrane localizations at EN. By contrast, DEGs of *lhycca1* are related to proteins targeted to the same cell compartments both at ED and EN. Thus, the clock components seem to regulate different time-of-day functions in all cell compartments.

### 2.6. Coordinated transcript and protein level changes reveal new points of clock regulation

**Figure 6.**
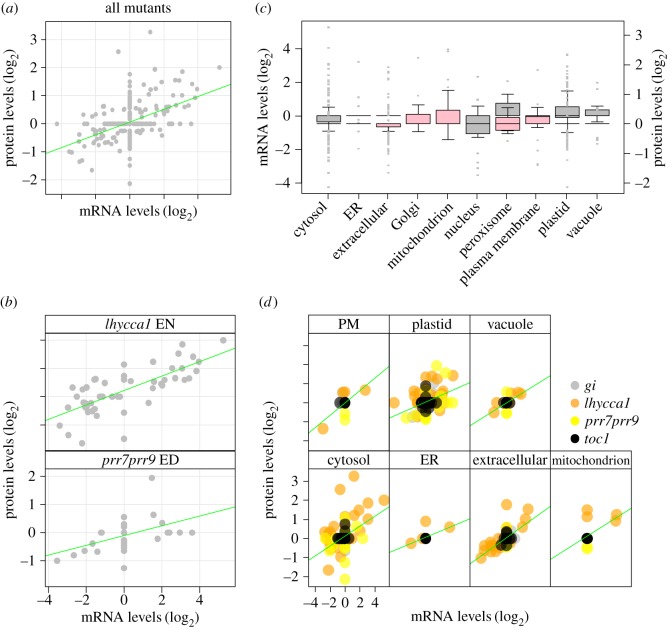
Correlation of genes exhibiting a coordinated change in transcript and protein and their corresponding subcellular localization. All log_2_-fold changes are relative to corresponding wild-type levels. (*a*) Linear regression analysis of the 51 genes encoding coordinately changing transcripts and proteins across all mutants ED and EN (*p* ≤ 2.00 × 10^−16^). (*b*) Correlated transcript–protein fluctuations in *lhycca1* EN (*p* = 7.05 × 10^−10^) and *prr7prr9* ED (*p* = 4.84 × 10^−4^). (*c*) Changes in transcript levels (shown in grey; primary *y*-axis) and protein (shown in pink; secondary *y*-axis) levels grouped by subcellular localization. (*d*) Linear regression analysis of the identified 51 genes encoding proteins localized to the plastid (*p* = 3.86 × 10^−5^), cytosol (*p* = 3.52 × 10^−5^), mitochondrion (*p* = 2.93 × 10^−2^), ER (*p* = 1.65 × 10^−2^), plasma membrane (*p* = 7.24 × 10^−3^), vacuole (*p* = 2.02 × 10^−03^) and extracellular (*p* = 7.23 × 10^−12^) across all four circadian clock mutants. Remaining compartments were not significant (electronic supplementary material, figure S4). PM, plasma membrane; ER, endoplasmic reticulum.

Using our datasets we next analysed how many genes have coordinated changes in their transcript and protein abundance. Coordinate changes are defined by either parallel or opposing increase or decrease in transcript and protein levels. Of all DEGs (FDR-adjusted *p* ≤ 0.05; more than 1.5-fold change), only 51 also showed a change in protein abundance (FDR-adjusted *p* ≤ 0.05; figure 6; electronic supplementary material, tables S14–S15). Linear regression analysis found significant correlation among the 51 transcript-protein pairs across all mutant backgrounds (*p* = 2.0 × 10^−16^, figure 6*a*). In particular, there was a strong correlation between transcript and protein levels in *lhycca1* at EN (*p* *=* 7.05 × 10^−10^) and *prr7prr9* at ED (*p* = 4.84 × 10^−04^; figure 6*b*; electronic supplementary material, figure S4). Of the 51 transcript-protein pairs, only two genes maintained a coordinated change in more than one experimental condition. These were a lipid transfer protein (AT4G22485) changing in *lhycca1* ED and EN, and a calcium binding family protein (AT2G41090) changing in *lhycca1* EN and *prr7prr9* ED. These results indicate that changes in the transcriptome of clock mutants are generally not reflected at the proteome level for those DEGs with quantitatively measured proteins. However, many of the DEGs belong to functional categories whose proteins are typically low abundant and consequently underrepresented in proteome analyses (such as TFs, kinases and phosphatases; figure 3). Furthermore, mutations in the circadian clock shift the ZT expression peak of clock-regulated transcripts [6,7]. This may also cause a shift in transcript oscillation, but the amplitude of expression and consequently the total amount of mRNA available for protein synthesis over the diurnal cycle could be stable. It is therefore possible that a large fraction of DEGs may have no impact on protein abundance. These possibilities can only be resolved through a more fine-grained analysis of diurnal transcript and protein levels in each clock mutant according to their specific period shift.

We used SUBAcon to assign the 51 transcript–protein pairs to their cell compartments (electronic supplementary material, table S16). Linear regression analysis showed a significant correlation between transcript and protein levels for the plastid (*p* = 3.86 × 10^−5^), cytosol (*p* = 3.52 × 10^−5^), mitochondrion (*p* = 2.93 × 10^−2^), ER (*p* = 1.65 × 10^−2^), plasma membrane (*p* = 7.24 × 10^−3^), vacuole (*p* = 2.02 × 10^−3^) and extracellular (*p* = 7.23 × 10^−12^) compartments (figure 6*c*,*d*; electronic supplementary material, figures S5–S6 and table S16). Thirty-five of these 51 transcript–protein pairs were found in *lhycca1* at EN, of which 14 encode proteins targeted to the cytosol and 11 to the ER/extracellular compartment. Only 10 transcript–protein pairs were coordinately changed in *lhycca1* at ED, which differed from those changing EN. The identification of coordinately changing transcript–protein pairs in *lhycca1* assigned to the ER/extracellular compartment is interesting because the ER has not been previously implicated in clock regulation. Clock control of the ER is not unexpected, however, considering its central role in Ca^2+^ signalling [90], protein maturation and secretion [91], as well as lipid [92] and hormone [92–94] metabolism (e.g. auxin).

Using the plant interactome database we retrieved known interacting partners of the ER-assigned proteins. At1g17860 and At4g31500 (CYP38B1) have been reported to interact with NODULARIN 26-LIKE MAJOR INTRINSIC PROTEIN 1;1 (NIP1;1) and GA INSENSITIVE DWARF 1A (GID1A), respectively (*Arabidopsis* Interactome Mapping Consortium, 2011). At1g54010, At3g16370 and At3g13750 each interact with the receptor-like kinase VASCULAR HIGHWAY 1/BRI1-LIKE 2 (VH1/BRL2; At2g01950), which is involved in both auxin and brassinosteroid signalling [95]. Considering the large number of DEGs in *lhycca1* related to auxin and ethylene metabolism and signalling, these results indicate potential connections between the hormone-related processes of the ER and the clock. Furthermore, genes At5g49360, At5g57655 and At3g54400 have been implicated in carbohydrate metabolism- and cell-wall-related processes, and may represent novel points of integration between the clock and the ER that require further investigation.

### 2.7. The LHY/CCA1 core clock proteins are most extensively connected to transcription factors and metabolism

The double mutant *lhycca1* has the most extensive changes in its transcriptome and proteome. GO categories related to isoprenoids (e.g. secondary metabolic process, GO:19 748; photosynthesis, GO:15 979; pigment biosynthetic process; GO:46 148), hormones (e.g. cellular response to hormone stimulus, GO:32 870; cellular response to abscisic acid stimulus, GO:71 215) and starch biosynthesis/degradation (e.g. carboxylic acid metabolic process, GO:19 752) were significantly over-represented among the DEGs. These GOs were further refined using curated information from multiple databases to create specific categories and maximize analysis depth of key signalling and metabolism processes (see Material and methods).

**Figure 7.**
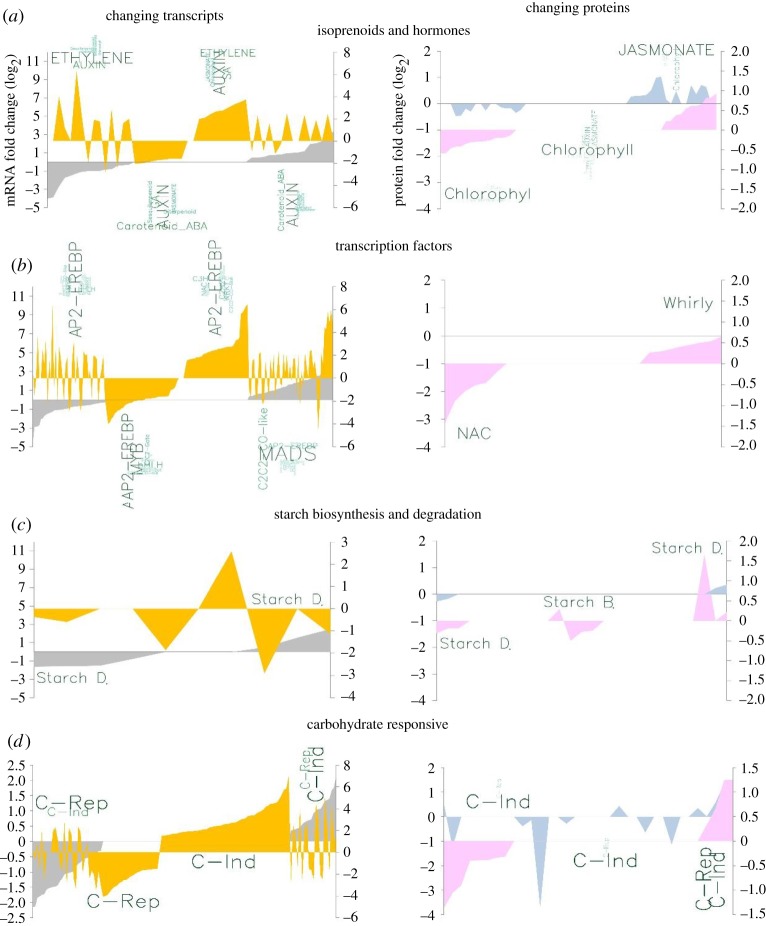
Targeted analysis of the changing *lhycca1* mutant transcriptome and proteome ED or EN. Fold change in transcript at ED (grey)/EN (yellow) and protein at ED (blue)/EN (pink). All changes are relative to wild-type levels. Each *x*-axis peak represents a gene outlined in electronic supplementary material, table S17. The *y*-axis (left) shows ED fold changes and *y*-axis (right) EN fold changes. Word clouds (green) above each gene set depict the changing gene families, pathways or cellular processes within a changing gene set (e.g. ‘MADS’ or ‘Chlorophyll’). Word size correlates with the frequency of the respective term. Categories examined include (*a*) isoprenoids and hormones, (*b*) transcription factors, (*c*) starch biosynthesis and degradation starvation, and (*d*) carbohydrate responsive (Starvation).

Across all categories for transcript–protein pairs, transcript abundance was more frequently and strongly affected than protein abundance both at ED and EN (figure 7). Moreover, changes in transcript abundance relative to wild-type levels often showed an opposite trend at ED versus EN, which can be explained by a ZT shift in gene expression peaks [18]. Consequently, transcripts that have LHY- and/or CCA1-controlled peak expression around dawn will peak late at night in *lhycca1*. The opposite pattern can be expected for transcripts with evening-phased peak expression, which would shift into the late day. Inverse abundance patterns at ED and EN were not generally found for the quantitatively measured proteins (figure 7). This may be attributed to the greater overall stability of proteins, by which diurnal oscillations in transcript abundance are integrated over longer periods of time at the protein level [61].

Figure 7 shows that metabolic pathways and cellular responses previously linked to the circadian clock are strongly affected in *lhycca1*. In particular, within the ‘isoprenoids and hormones’ category DEGs related to auxin metabolism and signalling have changed transcript levels at both ED and EN, while ethylene-related genes are mostly downregulated at ED (figure 7*a*; electronic supplementary material, table S17) [27,28,93]. At the protein level, genes encoding chlorophyll biosynthesis- and jasmonate-related proteins were most strongly enriched (figure 7*a*).

The category ‘transcription factors’ contains genes for which either transcript or protein abundance changes were found in *lhycca1*. Of the *Arabidopsis* TF families, AP2-EREBP (APETALA 2/ETHYLENE RESPONSIVE ELEMENT BINDING PROTEIN), MYB and MADS-box were those changing at the transcript level (figure 7*b*; electronic supplementary material, table S17). AP2-EREBP family TFs were enriched among both upregulated and downregulated transcripts at EN as well as among downregulated transcripts at ED. These TFs are often part of regulatory networks integrating light, sugar sensing and hormonal (ABA, auxin, ethylene) signals with retrograde signalling [96] or abiotic stress [97]. MYB proteins represent a large family of TFs in *Arabidopsis* that includes LHY and CCA1 [8,9]. Many MYB TFs regulate plant development and metabolism, such as phenylpropanoid biosynthesis [98], which is prominent in the clock mutant transcript and protein data (figure 3). MADS-box TFs also had transcript level changes in *lhycca1* plants. Many MADS-box TFs have been connected to plant development [99] and some to the clock through the family member SHORT VEGETATIVE PHASE (SVP), which represses flowering through FLOWERING LOCUS C (FLC) [85]. Changes among the TFs identified at the protein level affected NAC (AtCBF5; At3g57150) and WHIRLY (AtWHY1; At1g14410 and AtWHY3; At2g02740) family members as well as TFs of unknown function. WHIRLY TFs have been generally characterized as regulating defence responses [100], in addition to roles such as delaying senescence (AtWHY1 [101]) and maintaining plastid genome stability (AtWHY1/3 [58]). NAC TFs, however, are involved in plant development and stress responses [102], with AtCBF5 responsible for regulating *Arabidopsis* telomeres [103].

The categories ‘starch biosynthesis and degradation’ and ‘carbohydrate responsive’ were treated separately because starch biosynthesis and degradation are regulated independent of cellular carbohydrate response genes [69]. Gene expression changes related to starch biosynthesis and degradation are limited in *lhycca1* (figure 7*c*; electronic supplementary material, table S17), the mRNA encoding GRANULE BOUND STARCH SYNTHASE 1 (GBSS; At1g32900) was upregulated 2.5-fold at ED and downregulated 1.2-fold at EN. Similarly, mRNA levels for the three ß-amylases BAM4 (At5g55700), BAM6 (At2g32290) and BAM9 (At5g18670) also showed significant changes (electronic supplementary material, table S16); however, the exact function of these three BAM proteins is largely unknown. While *GBSS* and *BAM9* mRNA levels are circadian regulated with peak expression at EN, *BAM4* and *BAM6* mRNAs show no significant diurnal fluctuations (Diurnal DB)

The category ‘carbohydrate responsive’ contained genes that were shown to respond to changes in intracellular carbohydrate level but are not affected by the circadian clock ([69] and this report). In contrast with ‘starch biosynthesis and degradation’, the levels of many transcripts and proteins in the category ‘carbohydrate responsive’ were changed in *lhycca1* (figure 7*d*; electronic supplementary material, table S17). These changes are most pronounced at EN when ‘carbohydrate repressed’ transcripts and proteins are induced and ‘carbohydrate induced’ transcripts and proteins are strongly downregulated, indicating low intracellular carbohydrate levels. This is consistent with the carbon-starved status of *lhycca1* at EN [23,70]. Changes in carbohydrate responsive gene transcripts and proteins are also observed at ED albeit the number of genes affected and the amplitude of the changes is much lower compared to EN (figure 7*d*; electronic supplementary material, table S17). Rather than pointing towards reduced carbohydrate availability in *lhycca1* at ED this result is probably a consequence of the strong starvation response induced in the mutant plants at EN.

## 3. Conclusion

The results of the parallel analysis of transcriptome and proteome data from four *Arabidopsis* circadian clock mutants reveals that the core clock, morning and evening loops function in a modular fashion to regulate a significant portion of both the transcriptome and proteome. Furthermore, the detection of only 51 concurrently changing transcripts and proteins across all clock mutants suggests that there is only a limited coupling of transcript and protein regulation when the core clock is disrupted. This may be partially explained by the sampling of only two time points (ED and EN). However, clock control of cellular processes probably also involves other mechanisms, such as PTM of proteins and/or protein turnover, in addition to regulation of gene transcription [56,61,63]. Support for this hypothesis includes the large number of deregulated genes encoding protein kinases and ubiquitin ligases in all clock mutants, as well as the large number of phosphoproteins maintaining cyclical changes in a free-running cycle [55]. Considering the central role of PTMs in regulating cellular processes, further investigation of how PTMs regulate the circadian clock directly and influence its inputs and outputs is essential. Lastly, since the clock loops regulate discrete gene sets encoding proteins targeted to different subcellular compartments, it will be important to understand the impact of a compromised clock on physiological and metabolic processes represented by particular sub-proteomes under different photoperiod conditions.

## 4. Material and methods

### 4.1. Plant material and growth conditions

Clock mutants timing of cab expression (toc1-101; At5g61380 [104]), gigantea (gi.201; At1g22770 [58]), pseudo-response regulator 7/9 (prr7.3/prr9.1; At5g02810/At2g46790 [32]) and late elongated hypocotyl/circadian clock associated 1 (lhy-21/cca1-11; At1g01060/At2g46830 [105,106]) were provided by Prof Andrew Millar (University of Edinburgh, UK). *Arabidopsis* WT accessions Col-0 and WS-2 were obtained from common institute seed stock from Dr Karin Köhl (MPI for Molecular Plant Physiology, Potsdam). The toc1-101, gi.201 and prr7.3/prr9.1 mutants are in the Col-0 background and lhy-21/cca1-11 is in WS-2 background. Seeds were sown on wet soil in 10 cm diameter pots, covered with transparent lids and transferred to growth chambers (Percival Scientific Inc., Perry, IA, USA). Plants were grown in 12 L : 12 D cycles with light intensity controlled at 160 µmol m^−2^ s^−1^. The temperature was maintained at 20°C during the light phase and 18°C during the dark phase. Pots were randomized to decrease the impact of positional effects. After a week, the lids were removed and excess plants were thinned. On the 21st day after sowing on soil two to three biological replicates per time point were harvested. Each replicate consisted of a pool of six to nine plants. Sampling was performed at EN (ZT0) and ED (ZT12). Whole rosettes were cut at ground level, placed in plastic scintillation vials and frozen in liquid nitrogen.

### 4.2. Library preparation for RNA-sequencing

Total RNA was extracted from 33 samples with RNeasy Plant Mini Kit (Qiagen) following the manufacturer's protocols. The quality of the isolated RNA was determined with a Qubit (1.0) Fluorometer (Life Technologies, California, USA) and a Bioanalyzer 2100 (Agilent, Waldbronn, Germany). Only those samples with a 260/280 nm ratio between 1.8–2.1 and a 28S/18S ratio within 1.5–2 were further processed. The TruSeq RNA Sample Prep Kit v2 (Illumina, California, USA) was used in the succeeding steps. Briefly, total RNA samples (2500 ng) were poly A enriched and then reverse-transcribed into double-stranded cDNA. The cDNA samples was fragmented, end-repaired and polyadenylated before ligation of TruSeq adapters containing the index for multiplexing fragments containing TruSeq adapters on both ends were selectively enriched with PCR. The quality and quantity of the enriched libraries were validated using Qubit (1.0) Fluorometer and the Caliper GX LabChip GX (Caliper Life Sciences, USA). The product is a smear with an average fragment size of approximately 260 bp. The libraries were normalized to 10 nM in Tris–Cl 10 mM, pH 8.5 with 0.1% Tween 20.

### 4.3. Cluster generation and sequencing

The TruSeq PE Cluster Kit v3-cBot-HS or TruSeq SR Cluster Kit v3-cBot-HS (Illumina) was used for cluster generation using 10 pM of pooled normalized libraries on the cBOT. Sequencing were performed on the Illumina HiSeq 2500 single-end at 1 × 100 bp using the TruSeq SBS Kit v3-HS (Illumina).

### 4.4. RNA-sequencing read processing

Illumina genome analyser pipeline software CASAVA (v. 1.8.2) was used for image analysis, base calling and FASTQ file generation. The CLC Genomics Workbench 6 (http://www.clcbio.com) was used to perform the quality check analysis and to map RNA-seq reads to the custom *Arabidopsis* TAIR10_chr_rbr12c reference genome built in-house. TAIR10_chr_rbr12c was created from the TAIR10_genome release generic feature format version 3. The quality of the RNA-seq reads was verified using FASTQC software (www.bioinformatics.babraham.ac.uk/projects/fastqc/) integrated in the CLC Genomics Workbench 6 with the following parameters: quality limit: 0.05, remove 5′ terminal nucleotides: 3 and remove 3′ terminal nucleotides: 5. The mapping to the reference genome was performed using the following settings: maximum number of mismatches allowed: 2, mismatch cost: 2, insertion cost: 3, deletion cost: 3, minimum length fraction: 0.9, minimum similarity fraction: 0.8, unspecific match limit: 10, non-specific match handling: map randomly. As expression measure, the TOTAL_EXON_COUNT (i.e. the number of reads mapped to exons of the gene) for each sample was used for subsequent analyses. All RNA-seq data can be found on the Sequence Read Archive at NCBI (http://www.ncbi.nlm.nih.gov/sra; SRP082192).

### 4.5. RNA-seq data processing and differential expression analysis

The DESeq package [107] implemented in R (v. 3.1.0; R Core Team, 2015) was used for normalization and quantification of gene differential expression. The count tables for each sample comprised of the TOTAL_EXON_COUNT data exported from CLC Genomics Workbench. A non-specific pre-filtering step was conducted to filter out genes with less than two counts per million reads present in at least three samples. Differences in RNA composition for each library were taken into account through the normalization step implemented in the DESeq package [107]. The dispersion parameters for each transcript were estimated and a negative binomial generalized log-linear model was fitted to each gene read counts to assess the differential expression and to perform gene-wise statistical tests for the coefficient contrasts of interest (i.e. between mutant and control samples). All mutants and controls were measured as biological triplicates except for *prr7/prr9* at the ED and EN for which the available plant material limited the replication to two and one biological replicates, respectively. The DEGs between the *toc1*, *gi201* and *lhy/cca1* mutants and their corresponding wild-type control plants (Col-0 and Ws) at the ED and EN were identified as described above. The analysis of differential expression for *prr7prr9* at the ED and EN samples relative to their corresponding wild-type controls (Col-0) was performed separately. In brief, to estimate variability in the absence of true biological replicates, the available samples were treated as replicates of the same condition (i.e. *estimateDispersions(data, method* *=* ‘*blind*’, *sharingMode* *=* ‘*fit-only*’)) and the differential expression was assessed using the DESeq package [107]. As expected in the absence of biological replicates, fewer DEGs were identified. The overlap of the significantly changing transcripts (FDR-adjusted *p* ≤ 0.05 and log_2_-fold change threshold of 1.5) and proteins (FDR-adjusted *p* ≤ 0.05 and no log2-fold change threshold) for all mutants was calculated and displayed as Venn diagrams.

### 4.6. Protein extraction and digestion

Powdered frozen plant material (50 mg) was suspended in 100 µl SDS extraction medium (4% w/v SDS, 40 mM Tris, 60 µl ml^−1^ protease inhibitor cocktail (Roche)), mixed vigorously and centrifuged for 10 min at 16 000*g*. The supernatant was further centrifuged at 100 000*g* for 45 min. The resulting supernatant was diluted 4 : 1 (v/v) in Laemmli sample buffer and incubated at 65°C for 5 min. Approximately, 350 µg protein per sample was subjected to electrophoresis on a 10% SDS-polyacrylamide gel at 50 V overnight. Samples were loaded randomized on the gels to minimize positional effects. On each gel two standard wild-type extracts were run to allow normalization of data between gels. Gels were stained for 45 min in Coomassie Blue solution (20% v/v methanol, 10% v/v acetic acid, 0.1% m/v Coomassie Brilliant Blue R) then twice de-stained in 10% v/v methanol, 5% v/v acetic acid for 1 h at room temperature. Each lane of the gel was cut into seven fractions and transferred to a 96 deep well plate. Volumes of solutions were adjusted to ensure that the gel pieces were fully covered during the reduction, alkylation and washing steps. Gel pieces were washed three times with 50% v/v methanol, 100 mM ammonium bicarbonate, incubating each time for 1 h at 37°C. In-gel digestion of proteins using trypsin was performed as previously reported [108]. Volumes of solutions were adjusted to ensure that the gel pieces were fully covered during the reduction, alkylation and washing steps. After in-gel tryptic digestion peptides were purified by reversed-phase chromatography on Finisterre C18 SPE columns (Teknokroma, Barcelona, Spain) and dried at 45°C in a vacuum centrifuge.

### 4.7. Mass spectrometry analysis

Peptides were re-suspended in 40 µl 3% v/v acetonitrile, 0.1% v/v formic acid. Measurements were performed on a LTQ-Orbitrap XL (Thermo Scientific) coupled with a NanoLC 1D (Eksigent). Samples were loaded onto a laboratory-made capillary column (75 µm inner diameter, 9 cm long), packed with Magic C18 AQ beads (3 µm, 100 Å, Microm) at flow 0.5 µl min^−1^ in 3% v/v acetonitrile, 0.2% v/v formic acid and eluted with a 5–40% v/v acetonitrile concentration gradient over 70 min, followed by 80% v/v acetonitrile for 10 min, at 0.25 µl min^−1^. Peptide ions were detected in a full scan from mass-to-charge ratio 300–2000. MS/MS scans were performed for the five peptides with the highest MS signal (minimal signal strength 500 hits, isolation width mass-to-charge ratio 3 *m*/*z*, relative collision energy 35%). Peptides for which MS/MS spectra had been recorded were excluded from further MS/MS scans for 20 s.

### 4.8. Peak area based protein quantification and statistical analysis

Quantitative analysis of MS/MS measurements was performed with Progenesis LCMS software (Nonlinear Dynamics). The full *m*/*z* range was imported; the peak picking time was limited to between 20 and 80 min of the LCMS run. One run was selected as a standard and for each run 15 vectors were placed manually on prominent peaks before applying the automatic alignment function of Progenesis, followed by the peak picking function. Progenesis-calculated normalization factors, all between 0.8 and 1.2, were applied across the runs. Following this, the best six spectra for each peak were exported to Mascot. Mascot search parameters were set as follows: *Arabidopsis* TAIR10 genome annotation, requirement for tryptic ends, one missed cleavage allowed, fixed modification: carbamidomethylation (cysteine), variable modification: oxidation (methionine), peptide mass tolerance = ±1.2 Da, MS/MS tolerance = ±0.6 Da, allowed peptide charges of +2 and +3. Spectra were also searched against a decoy database of the *Arabidopsis* proteome. Search results were filtered to ensure a FDR below 1% and identifications with a Mascot score below 25 were excluded. Mascot results were imported into Progenesis, quantitative peak area information extracted and the results exported for data plotting and statistical analysis. All mutants and controls were measured as biological triplicates except for *prr7/prr9* EN for which the available plant material limited analysis to one biological replicate. Mass spectrometry data used for quantitation can be found in electronic supplementary material, table S18 as well as on the EMBL proteomic repository PRoteomics IDEntifications (PRIDE; https://www.ebi.ac.uk/pride/archive/; 10.6019/PXD004763). Significant changes in protein abundance for each mutant compared to their respective wild-type background were determined by ANOVA and adjusted for multiple testing using the Benjamini & Hochberg method [109]. This analysis was performed in R (v. 3.2.3; R Core Team, 2015).

### 4.9. Subcellular localization, gene ontology analysis and over-representation analysis

Subcellular localization of proteins and translated transcripts was determined using the *in silico* SUBAcon consensus subcellular localization predictor housed in the *Arabidopsis* Subcellular Database—SUBA v. 3 [110] (http://suba.plantenergy.uwa.edu.au/). The GO analysis was performed using the BinGO software [111] implemented in Cytoscape [112] and was displayed using REVIGO [113]. Based on the top 25 significantly over-represented GOs (hypergeometric test *p* ≤ 0.05) and additional classes (e.g. TFs, PTM), detailed categories were compiled and used for a focused over-representation analysis. The genes for each compiled category were retrieved from partly or entirely manually curated public databases: isoprenoids and hormones—AtIPD [114] (http://www.atipd.ethz.ch/); starch biosynthesis and degradation—MapMan [115] (http://mapman.gabipd.org/) and GSEA plants [116] (http://structuralbiology.cau.edu.cn/PlantGSEA/), carbon responsive genes [69], cold responsive genes—MapMan [115] (http://mapman.gabipd.org/), protein kinases and protein phosphatases—AthKD (http://bioinformatics.cau.edu.cn/athKD/index.htm), TFs—PlnTFDB [117] (http://plntfdb.bio.uni-potsdam.de/v3.0/), RARTF [118] (http://rarge.psc.riken.jp/rartf/), DATF [119] (http://datf.cbi.pku.edu.cn/), AtTFDB [120] (http://arabidopsis.med.ohio-state.edu/AtTFDB/) and other PTM modifiers from TAIR (https://www.arabidopsis.org/). The over-representation of the DEGs for each compiled category was evaluated using the Fisher's exact test implemented in R (v. 3.1.0; R Core Team, 2015).

## Supplementary Material

Complete Supplemental Figure Listing

## Supplementary Material

Figure S1

## Supplementary Material

Figure S2

## Supplementary Material

Figure S3

## Supplementary Material

Figure S4

## Supplementary Material

Figure S5

## Supplementary Material

Figure S6

## Supplementary Material

Complete Supplemental Table Listing

## Supplementary Material

Table S1

## Supplementary Material

Table S2

## Supplementary Material

Table S3

## Supplementary Material

Table S4

## Supplementary Material

Table S5

## Supplementary Material

Table S6

## Supplementary Material

Table S7

## Supplementary Material

Table S8

## Supplementary Material

Table S9

## Supplementary Material

Table S10

## Supplementary Material

Table S11

## Supplementary Material

Table S12

## Supplementary Material

Table S13

## Supplementary Material

Table S14

## Supplementary Material

Table S15

## Supplementary Material

Table S16

## Supplementary Material

Table S17

## Supplementary Material

Table S18
