# Supplementary material for: Parallel analysis of Arabidopsis circadian clock mutants reveals different scales of transcriptome and proteome regulation
Source: Open Biol. 2017 Mar 1;7(3):160333. doi: 10.1098/rsob.160333 (PMC5376707; doi:10.1098/rsob.160333)
Supplement: Complete Supplemental Figure Listing [file rsob160333supp1.pdf]

SUPPLEMENTAL FIGURES

Figure S1. GO analysis of transcripts and proteins differentially expressed in Col-0 and Ws2 wild-type Arabidopsis ED and EN.

Figure S2. Example distribution of the transcript fold change values of the differentially expressed genes.

Figure S3. Circadian clock *cis*-regulatory elements located in the first 2000bp upstream protein kinase and ubiquitin ligase genes significantly changing at the transcript level (FDR corrected *p-value* ≤ 0.05 and FC ≥ 1.5).

Figure S4. Linear regression analysis of the 51 genes whose transcripts and protein exhibit a correlative changes in abundance.

Figure S5. Fold Changes in the 51 genes maintaining concurrently changing transcripts and proteins in the lhycca1 mutant EN grouped by subcellular localization.

Figure S6. Linear regression analysis of concurrently changing transcripts and proteins across different subcellular locations ED and EN.
