# Supplementary material for: Parallel analysis of Arabidopsis circadian clock mutants reveals different scales of transcriptome and proteome regulation
Source: Open Biol. 2017 Mar 1;7(3):160333. doi: 10.1098/rsob.160333 (PMC5376707; doi:10.1098/rsob.160333)
Supplement: Figure S1 [file rsob160333supp2.pdf]

# Supplementary Figure 1

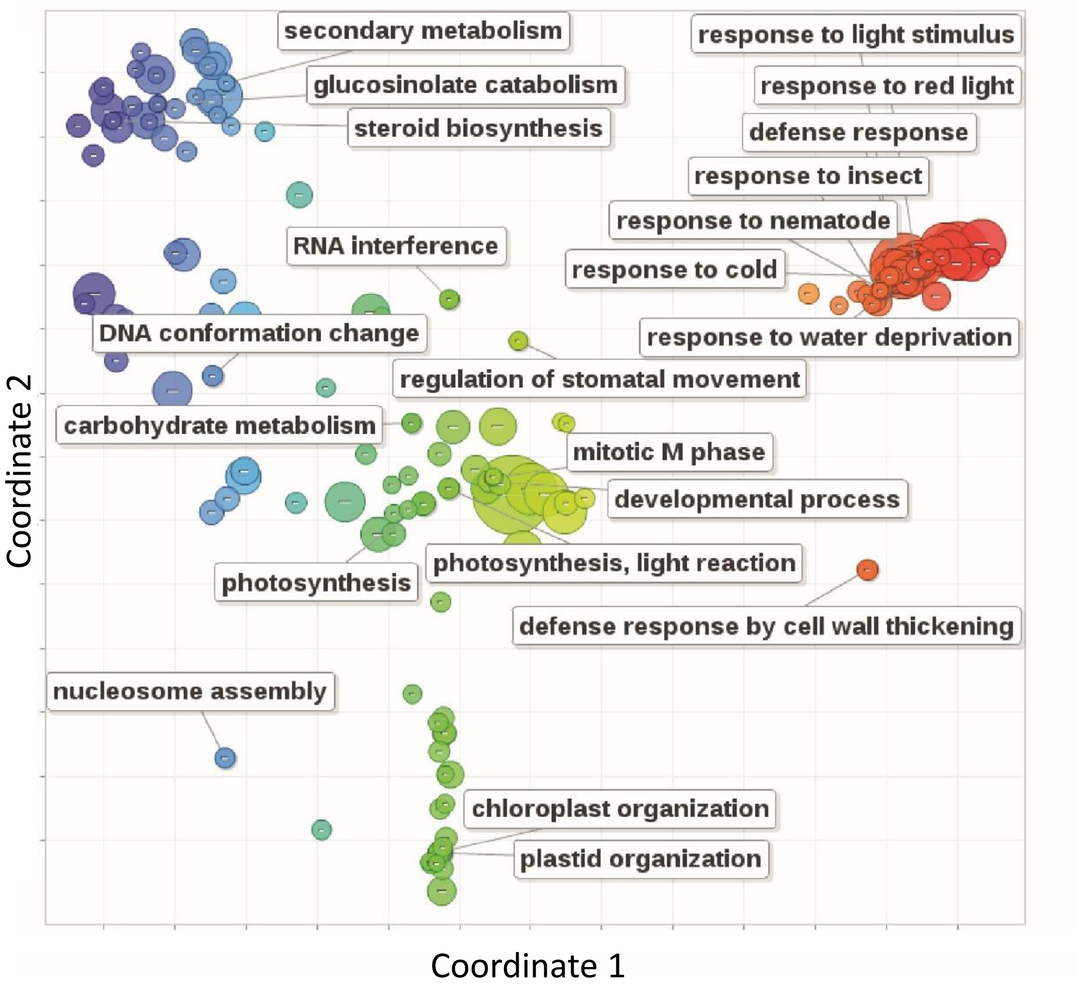


**Figure S1. REVIGO-based GO analysis of transcripts and proteins differentially expressed in Col-0 and Ws2 wild-type Arabidopsis ED and EN.** The scatterplot shows the unique GO terms in a two dimensional visualization space (coordinate 1 and 2) determined by multidimensional scaling of a matrix of the GO terms' semantic similarities such that more semantically similar GO terms are also closer in the plot. The colors represent the semantic similarity of the GO terms. The size of the circle corresponds to the *p-value* of the hypergeometric test applied to identify significant enrichments of GO terms (*p-value* $\leq 0.05$) in the list of deregulated genes (smaller *p-value* correspond to larger bubbles). The REVIGO analysis tool can be found at http://revigo.irb.hr [113].
