# Supplementary material for: Parallel analysis of Arabidopsis circadian clock mutants reveals different scales of transcriptome and proteome regulation
Source: Open Biol. 2017 Mar 1;7(3):160333. doi: 10.1098/rsob.160333 (PMC5376707; doi:10.1098/rsob.160333)
Supplement: Figure S2 [file rsob160333supp3.pdf]

Figure S2

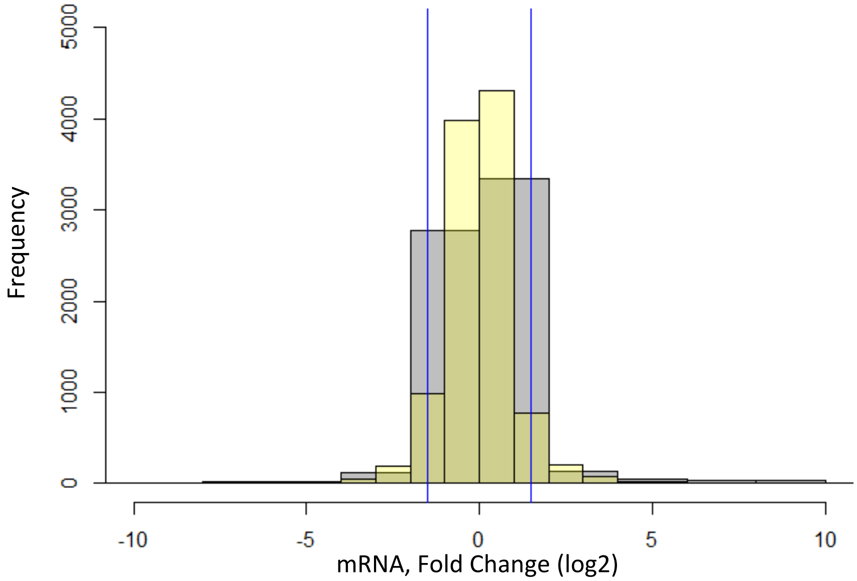

Figure S2. Example distribution of the transcript fold change values of the differentially expressed genes. Yellow - *lhycca1* EN and gray – Ws2 EN. The majority of the differentially expressed genes (FDR corrected *p-value* ≤ 0.05) maintained a fold-change lower than 1.5 and higher than -1.5, respectively (blue lines). 1105 genes were differentially expressed over the 1.5 fold threshold in the *lhycca1* mutant EN, representing 10% of all differentially expressed genes in this mutant.
