# Supplementary material for: Parallel analysis of Arabidopsis circadian clock mutants reveals different scales of transcriptome and proteome regulation
Source: Open Biol. 2017 Mar 1;7(3):160333. doi: 10.1098/rsob.160333 (PMC5376707; doi:10.1098/rsob.160333)
Supplement: Figure S3 [file rsob160333supp4.pdf]

Figure S3

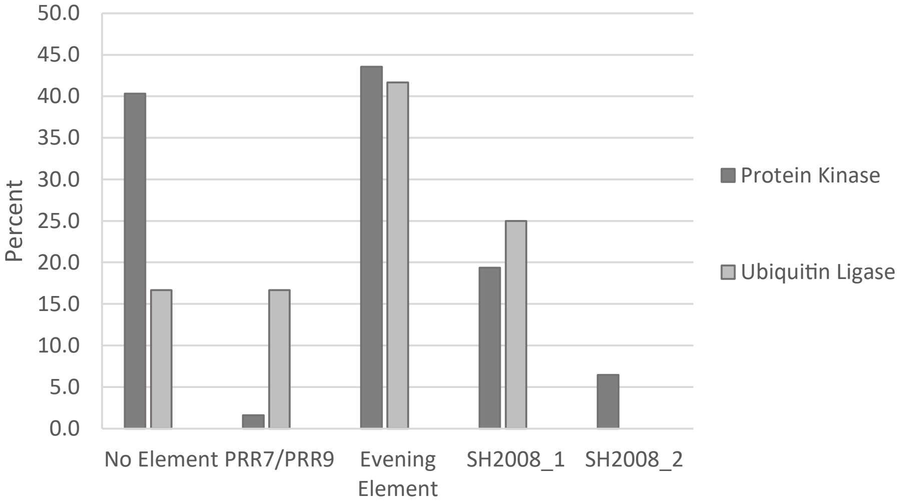

Figure S3. Circadian clock *cis*-regulatory elements located in the first 2000bp upstream protein kinase and ubiquitin ligase genes significantly changing at the transcript level (FDR corrected *p-value* ≤ 0.05 and FC ≥ 1.5). Percent of genes maintaining each element is presented. Circadian clock *cis*-regulatory elements were previously described [19, 68, 71]. Protein kinase and ubiquitin ligase gene information can be found in Table S8 along with the position of each element within each genes promoter.
