# Supplementary material for: Parallel analysis of Arabidopsis circadian clock mutants reveals different scales of transcriptome and proteome regulation
Source: Open Biol. 2017 Mar 1;7(3):160333. doi: 10.1098/rsob.160333 (PMC5376707; doi:10.1098/rsob.160333)
Supplement: Figure S4 [file rsob160333supp5.pdf]

Figure S4

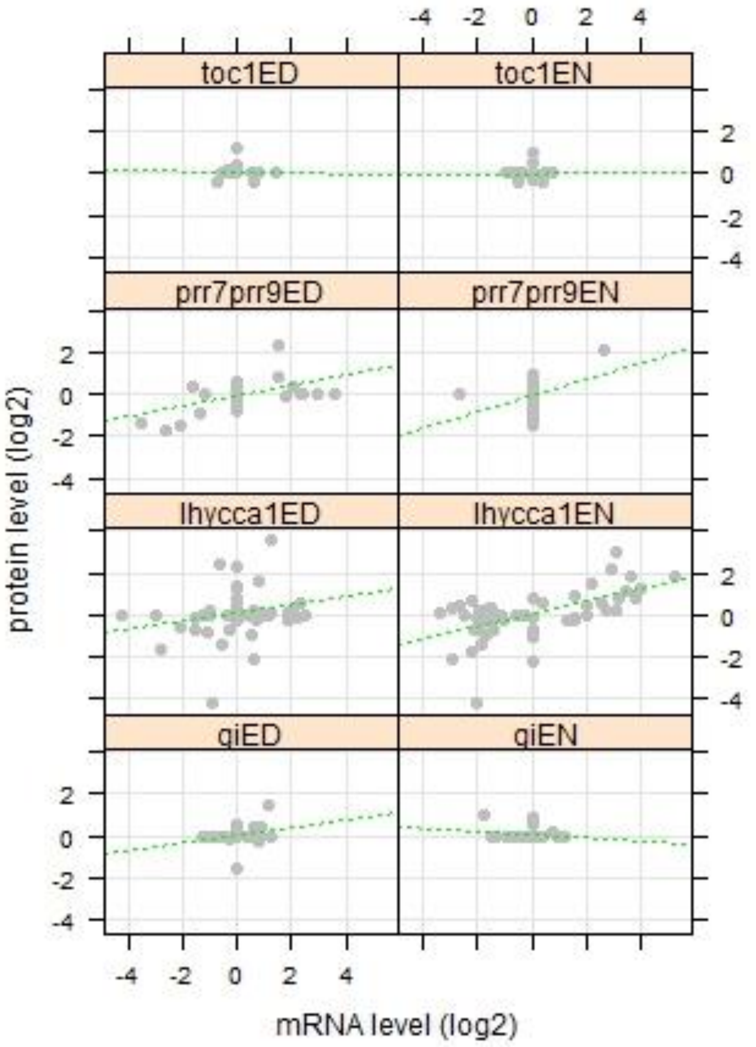

Figure S4. Linear regression analysis of the 51 genes whose transcripts and protein exhibit a correlative changes in abundance. Depicted are the transcripts (x-axis) and proteins (y-axis) of the 51 genes in each circadian clock mutant ED and EN. Green lines represent the linear regression. Correlated fluctuations were observed in the *lhycca1* mutant EN (*p-value* = $4.80e^{-06}$) and in the *prr7prr9* mutant ED (*p-value* = $5.09e^{-05}$) and EN (*p-value* = $5.10e^{-03}$).
