# Supplementary material for: Parallel analysis of Arabidopsis circadian clock mutants reveals different scales of transcriptome and proteome regulation
Source: Open Biol. 2017 Mar 1;7(3):160333. doi: 10.1098/rsob.160333 (PMC5376707; doi:10.1098/rsob.160333)
Supplement: Figure S5 [file rsob160333supp6.pdf]

Figure S5

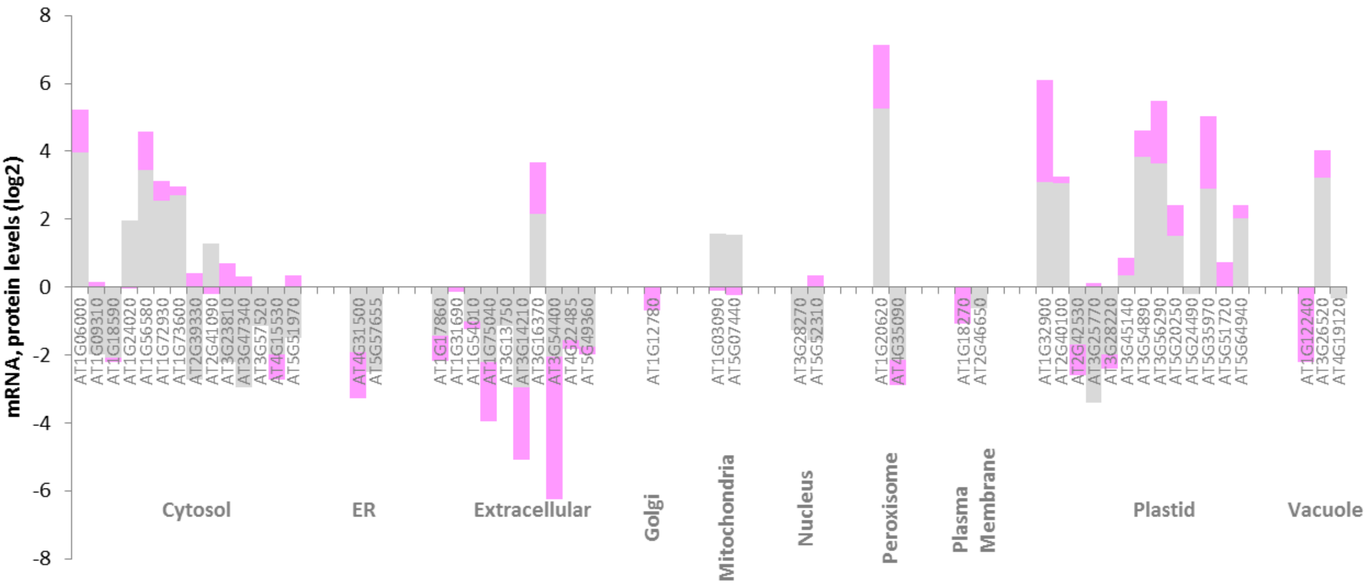

Figure S5. Fold changes in the 51 genes maintaining concurrently changing transcripts and proteins in the *lhycca1* mutant EN grouped by subcellular localization. Depicted in grey and pink are the changing transcripts and proteins, respectively. Subcellular localizations were defined using SUBAcon as described in the Materials and Methods.
