# Supplementary material for: Parallel analysis of Arabidopsis circadian clock mutants reveals different scales of transcriptome and proteome regulation
Source: Open Biol. 2017 Mar 1;7(3):160333. doi: 10.1098/rsob.160333 (PMC5376707; doi:10.1098/rsob.160333)
Supplement: Figure S6 [file rsob160333supp7.pdf]

Figure S6

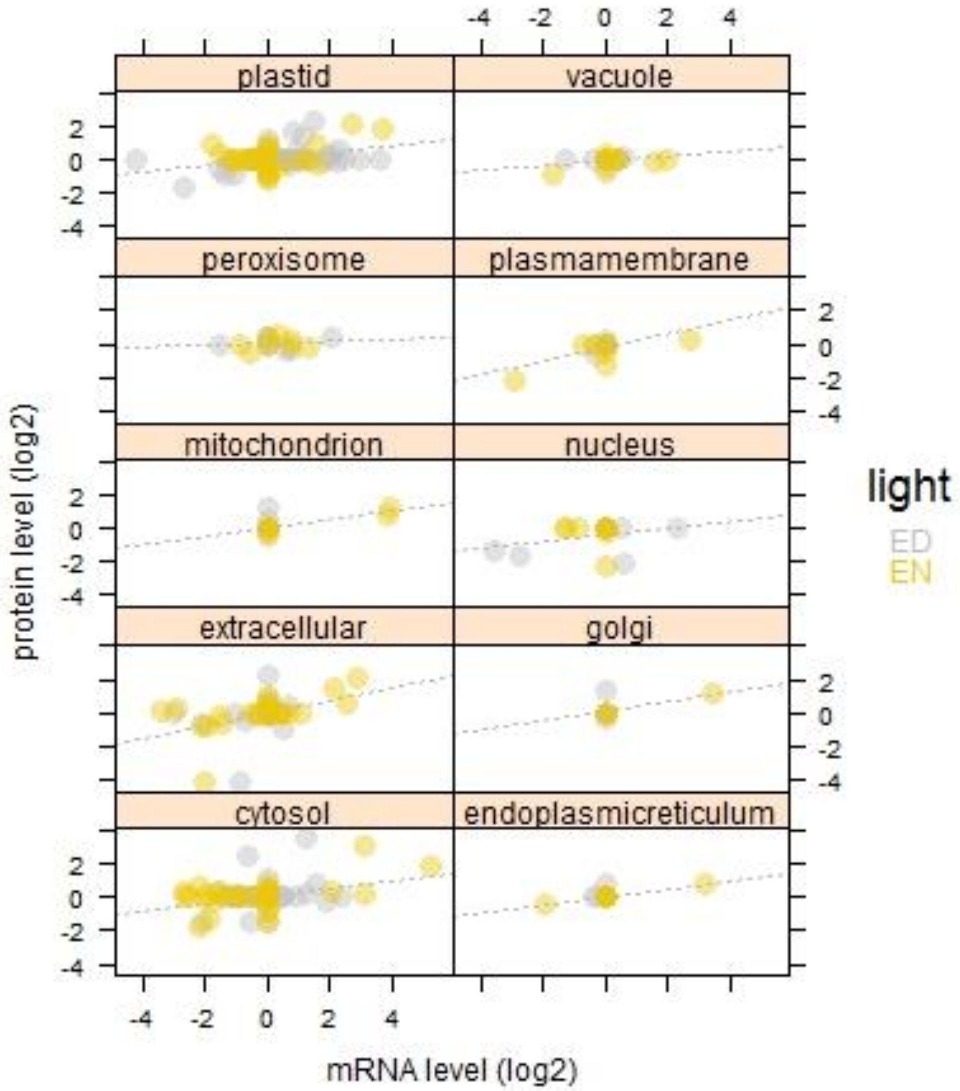

Figure S6. Linear regression analysis of concurrently changing transcripts and proteins across different subcellular locations ED and EN. Depicted are the transcripts (X-axis) and proteins (Y-axis) of the 51 genes in each circadian clock mutant ED and EN across several subcellular locations. Grey lines represent linear regressions. ED samples are shown in grey and EN in yellow.
