# Supplementary material for: Parallel analysis of Arabidopsis circadian clock mutants reveals different scales of transcriptome and proteome regulation
Source: Open Biol. 2017 Mar 1;7(3):160333. doi: 10.1098/rsob.160333 (PMC5376707; doi:10.1098/rsob.160333)
Supplement: Complete Supplemental Table Listing [file rsob160333supp8.pdf]

SUPPLEMENTAL TABLES

Table S1. Transcript data containing AGIs, FDR adjusted *P-value* and fold changes in abundance.

Table S2. Protein data containing AGIs, FDR adjusted *P-value* and fold changes in abundance.

Table S3. GO analysis depicting the comparison of Col-0 versus Ws transcriptome changes.

Table S4. Comparison of Col-0 and Ws transcript changes ED versus EN.

Table S5. Comparison of Col-0 and Ws proteome changes ED versus EN.

Table S6. Fisher's Exact Test for over-representation of significantly changing transcripts and proteins in the different mutants at ED or EN.

Table S7. Over-representation data for mRNA and proteins.

Table S8. Significantly changing carbohydrate responsive transcripts.

Table S9. Protein kinases and ligases showing a ≥1.5 fold-change in transcript abundance in across the circadian clock mutant population.

Table S10. Over-representation of small gene sets: mRNA. AGIs, compiled categories and gene annotation information provided.

Table S11. Over-representation of small gene sets: Protein. AGIs, compiled categories and gene annotation information provided.

Table S12. Consensus subcellular localization of all genes changing in abundance at the transcript level.

Table S13. Consensus subcellular localization of all genes changing in abundance at the protein level.

Table S14. Directionality of concurrently changing transcripts and proteins.

Table S15. List of genes exhibiting a correlative changes in transcript and protein abundance.

Table S16 Consensus subcellular localization of genes exhibiting both a change in transcript and protein abundance.

Table S17. Transcripts and proteins pertaining to isoprenoids / hormones, transcription factors and starch / starvation exhibiting an abundance change in *lhycca1* at ED or EN.

Table S18. Mass spectrometry data employed in label-free quantitation.
