# Supplementary material for: Parallel analysis of Arabidopsis circadian clock mutants reveals different scales of transcriptome and proteome regulation
Source: Open Biol. 2017 Mar 1;7(3):160333. doi: 10.1098/rsob.160333 (PMC5376707; doi:10.1098/rsob.160333)
Supplement: Table S6 [file rsob160333supp14.pdf]

Table S6

Table S6. Fisher's Exact Test for over-representation of significantly changing transcripts and proteins in the different mutants at ED or EN. The Mockler database (Diurnal DB) was used to extract genes with circadian and/or diurnal oscillations. Highlighted in green are those which possess significant over-representation ($p<0.05$).

	Count Circadian	Count Diurnal	Circadian Peak M	Circadian Peak E	Diurnal Peak M	Diurnal Peak E	Total Sign.	
lhycca1 ED	3.26E-122	3.19E-113	3.07E-42	5.73E-26	7.25E-37	1.57E-27	4448	Transcripts
lhycca1 EN	2.34E-16	6.59E-11	3.87E-04	1.83E-02	1.08E-05	2.77E-01	10658	
toc1 ED	2.96E-111	1.23E-66	9.25E-68	5.10E-02	7.10E-61	7.10E-01	942	
toc1 EN	2.78E-155	1.10E-87	1.52E-38	3.25E-05	1.47E-18	6.39E-04	1995	
prr79 ED	1.77E-74	5.55E-44	2.07E-13	4.81E-01	9.53E-13	2.93E-02	460	
prr79 EN	3.34E-01	6.32E-01	1.00E+00	1.00E+00	1.00E+00	3.26E-02	16	
gi ED	6.13E-122	4.20E-91	1.44E-27	1.86E-02	1.11E-24	3.86E-02	2166	
gi EN	3.79E-189	4.03E-158	5.57E-80	1.81E-27	7.07E-69	1.15E-15	1995	
lhycca1 ED	6.10E-12	3.58E-07	2.28E-02	2.93E-02	5.20E-01	2.03E-01	328	Proteins
lhycca1 EN	2.31E-03	1.54E-02	1.99E-01	1.60E-01	6.79E-01	2.29E-01	633	
toc1 ED	4.04E-03	6.35E-05	2.00E-02	3.43E-01	5.73E-03	1.59E-01	97	
toc1 EN	1.24E-02	2.02E-02	5.47E-02	9.65E-01	3.40E-01	7.81E-01	171	
prr79 ED	2.29E-10	2.35E-05	4.55E-02	7.77E-03	2.25E-01	1.44E-01	413	
prr79 EN	3.44E-02	2.29E-01	2.06E-01	4.76E-01	8.41E-01	2.52E-01	614	
gi ED	1.35E-02	2.03E-02	2.91E-01	2.68E-01	2.95E-01	9.39E-02	109	
gi EN	4.83E-03	2.91E-04	9.87E-02	7.29E-01	6.09E-01	2.42E-01	95	
totals T	3280	5814	485	409	766	803	0	
totals P	918	1386	129	125	104	226	0	

Count Circadian: Enrichment of circadian regulated transcripts
Count Diurnal: Enrichment of diurnally oscillating transcripts
Circadian and Diurnal Peak: Circadian and Diurnal transcripts separated according to the peak expression (M = morning, E = evening).
Total Sign: Total number of proteins/transcripts showing significant changes
Total T and P: Total number of transcripts/proteins falling into a particular category (row 1)
