# Supplementary material for: Parallel analysis of Arabidopsis circadian clock mutants reveals different scales of transcriptome and proteome regulation
Source: Open Biol. 2017 Mar 1;7(3):160333. doi: 10.1098/rsob.160333 (PMC5376707; doi:10.1098/rsob.160333)
Supplement: Table S14 [file rsob160333supp22.pdf]

Table S14

Table S14. Directionality of concurrently changing transcripts and proteins. Complements of clock related genes and carbon responsive genes were obtained from Diurnal DB and Usadel et al., 2008, respectively.

	Overlap Total	Same Dir.	Same Dir. Clock	Same Dir. Carbon	Opposite Dir.	Oppos. Dir. Clock	Oppos. Dir. Carbon	Total sign. T	Total sign. P
lhycca1 ED	10	8	5	0	2	1	0	287	328
lhycca1 EN	35	30	21	21	5	4	3	1105	633
toc1 ED	0	0	0	0	0	0	0	28	97
toc1 EN	0	0	0	0	0	0	0	61	171
prr79 ED	7	6	4	3	1	1	0	287	413
prr79 EN	1	1	1	0	0	0	0	16	614
gi ED	0	0	0	0	0	0	0	65	109
gi EN	1	1	1	1	0	0	0	64	95
