# Supplementary material for: Parallel analysis of Arabidopsis circadian clock mutants reveals different scales of transcriptome and proteome regulation
Source: Open Biol. 2017 Mar 1;7(3):160333. doi: 10.1098/rsob.160333 (PMC5376707; doi:10.1098/rsob.160333)
Supplement: Table S16 [file rsob160333supp24.pdf]

Table S16

Table S16. Consensus subcellular localization of genes exhibiting both a change in transcript and protein abundance. Consensus *in silico* subcellular localization prediction algorithm SUBAcon (See Materials and Methods) was used to assess the subcellular localization of genes exhibiting a significant and corresponding change in both mRNA transcript and protein levels either ED or EN.

mutant	No. Overlap Transcripts and Proteins	Subcellular Localization					
		Chloroplast (No.)	Cytosol (No.)	ER (No.)	Mitochondria (No.)	Nucleus (No.)	Peroxisomes (No.)
giED	0	na	na	na	na	na	na
giEN	1	1	0	0	0	0	0
lhycca1ED	10	2	2	2	0	0	2
lhycca1EN	35	2	14	11	2	1	1
prr7prr9ED	7	3	4	0	0	0	0
prr7prr9EN	1	1	0	0	0	0	0
toc1ED	0	na	na	na	na	na	na
toc1EN	0	na	na	na	na	na	na
